# Multi-Stage Classification of Retinal OCT Using Multi-Scale Ensemble Deep Architecture

**DOI:** 10.3390/bioengineering10070823

**Published:** 2023-07-10

**Authors:** Oluwatunmise Akinniyi, Md Mahmudur Rahman, Harpal Singh Sandhu, Ayman El-Baz, Fahmi Khalifa

**Affiliations:** 1 Department of Computer Science, School of Computer, Mathematical and Natural Sciences, Morgan State University, Baltimore, MD 21251, USA; olaki58@morgan.edu (O.A.); md.rahman@morgan.edu (M.M.R.); 2 Bioengineering Department, University of Louisville, Louisville, KY 20292, USA; harpal.sandhu@louisville.edu (H.S.S.); ayman.elbaz@louisville.edu (A.E.-B.); 3 Electronics and Communications Engineering Department, Mansoura University, Mansoura 35516, Egypt; 4 Electrical and Computer Engineering Department, Morgan State University, Baltimore MD 21251, USA

**Keywords:** ensemble learning, OCT, pyramidal network, feature fusion, scale-adaptive

## Abstract

Accurate noninvasive diagnosis of retinal disorders is required for appropriate treatment or precision medicine. This work proposes a multi-stage classification network built on a multi-scale (pyramidal) feature ensemble architecture for retinal image classification using optical coherence tomography (OCT) images. First, a scale-adaptive neural network is developed to produce multi-scale inputs for feature extraction and ensemble learning. The larger input sizes yield more global information, while the smaller input sizes focus on local details. Then, a feature-rich pyramidal architecture is designed to extract multi-scale features as inputs using DenseNet as the backbone. The advantage of the hierarchical structure is that it allows the system to extract multi-scale, information-rich features for the accurate classification of retinal disorders. Evaluation on two public OCT datasets containing normal and abnormal retinas (e.g., diabetic macular edema (DME), choroidal neovascularization (CNV), age-related macular degeneration (AMD), and Drusen) and comparison against recent networks demonstrates the advantages of the proposed architecture’s ability to produce feature-rich classification with average accuracy of 97.78%, 96.83%, and 94.26% for the first (binary) stage, second (three-class) stage, and all-at-once (four-class) classification, respectively, using cross-validation experiments using the first dataset. In the second dataset, our system showed an overall accuracy, sensitivity, and specificity of 99.69%, 99.71%, and 99.87%, respectively. Overall, the tangible advantages of the proposed network for enhanced feature learning might be used in various medical image classification tasks where scale-invariant features are crucial for precise diagnosis.

## 1. Introduction

Specialized non-invasive imaging techniques are extensively utilized in clinical research to detect/diagnose retinal diseases that may lead to vision loss. In practice, different image types are exploited for that purpose, including optical coherence tomography (OCT), fundus photography, OCT angiography (OCTA), etc. The OCT-based imaging technique in particular is widely exploited in clinical practice due to its ability to produce high-resolution cross-sectional images of the retina, which greatly help in the assessment of several retinal diseases [1,2]. However, due to the complexity and variability of the image features, accurate classification of OCT images is challenging. Developing an accurate diagnostic system for diseases is clinically essential for personalized medicine [3]. Furthermore, retinal disease diagnosis is a critical target since it is almost entirely subjective and the appropriate treatment path to effectively manage retina diseases relies on the accuracy of the diagnosis.

Retinal image diagnosis has shown an increased interest recently from various research groups. A large volume of research work has shown promising results in improving the accuracy and efficiency of OCT-based image analysis [4]. The accuracy of OCT image classification has shown considerable promise when using machine learning (ML) techniques. Particularly, the use of deep learning (DL) can optimize solutions to several complex classification problems [5]. DL-based techniques have the potential to perform efficient classification as well as segmentation of various structures (e.g., drusen) and grading of OCT images [6,7,8,9].

In recent years, several ML/DL research papers have been published on retinal image classification for various diseases, e.g., age-related macular degeneration (AMD), diabetic retinopathy (DR), diabetic macular edema (DME), and choroidal neovascularization (CNV). A few papers have proposed ensemble methods to improve the overall accuracy of retinal image classification tasks for macular diseases (e.g., AMD, CNV, DR, DME, etc.) by combining multiple DL models. For example, multi-step techniques for DR diagnosis using OCT were proposed by Elgafi et al. [10]. The system sequentially segments the retinal layers, extracts 3D retinal features, and uses a multilayer perceptron (MLP) for classification using the extracted features. In a leave-one-subject-out evaluation, their system achieved an accuracy of 96.81%. A similar approach with the addition of a feature selection step using the Firefly algorithm was proposed in Reference [11] by Özdaş et al. Multiple binary classifications were conducted using two public datasets and achieved a mean accuracy of 0.957 and 0.954, respectively. A multi-scale convolutional mixture of expert (MCME) ensemble models was proposed in Reference [12] by Rasti et al. to separate the normal retina from DME and dry AMD. The authors also introduced a new cost function for discriminative and fast learning. The system has been evaluated on a total of 193 subjects and demonstrated a precision rate and area under the curve (AUC) of 98.86% and 0.9985, respectively. Ai et al. [13] proposed a fusion network (FN)-based disease detection algorithm for retinal OCT images. They utilized InceptionV3, Inception-ResNet, and Xception DL algorithms as base classifiers, each accompanied by an attention mechanism. Multiple prediction–fusion strategies were employed to output the final prediction results. Comparison to other algorithms showed improved accuracy in the classification of the diseases. A shallow network of only five layers was introduced by Ara et al. in Reference [14] for OCT-B scan classification. The authors investigated the effects of image augmentation as well as deeper networks on final classification. The approach reduced computational time by 16.5% based on the model size, and data augmentation yielded improved accuracy.

A study by Tvenning et al. [15] utilized a DL-based method for AMD identification on OCT scans. The neural architecture, so-called OptiNet, integrates classical DL networks and different parallel layer-wise modules created from filter features. The systems have been evaluated on 600 AMD cases and documented the ability of the deep network to detect alterations in retinal scan regions that correspond to the retinal nerve fiber and choroid layers, which can be linked to AMD. Another CNN-based approach for macular disease classification was proposed by Mishra et al. [16]. the authors introduced a deformation-aware attention-based module to encode crucial morphological variations of retinal layers. The proposed module was integrated into a transfer-learning(TL)-based deep network. The main advantage of the proposed approach is that it is void of preprocessing steps, and the results showed superior performance over competing methods. Another attention-based architecture was proposed by Huang et al. in Reference [17]. Due to the ability of their global attention block (GAB) to focus on lesion locations in the OCTs, the authors proposed a lightweight classification network model. Evaluation on the public UCSD dataset has demonstrated superior classification compared to commonly used attention mechanisms. S.-Paima et al. [18] developed a two-stage multi-scale method for classifying AMD-related pathologies using different backbone models. Hierarchical features were extracted from the input images. This end-to-end model employed a single convolutional neural network (CNN) model to extract different-sized features which were then fused for classification. Two sets of datasets were used: 12,649 images from NCH and 108,312 images from UCSD [19]. Using pre-trained ImageNet weights, the model accuracy was 92.0% ± 1.6%, which was boosted 93.4% ± 1.4% in stage two by fine-tuning the model.

A multi-scale deep feature fusion (MDFF) approach was introduced by Das et al. [20]. The model leveraged the fusion of features from multiple scales—thereby capturing the inter-scale variations in images in order to introduce discriminative and complementary features—and employed transfer learning to reduce training parameters. TL, however, reduces dependence and has poor adaptation to the differences among different datasets. Similarly, Li et al. [21] used a deep TL-based method to fine-tune the pre-trained VGG-16 in order to classify 109,312 images and thereby obtained a prediction accuracy of 98.6%. The validation dataset was also used as the testing dataset, so the reported performance could be biased, and training the model on inadequate amounts of data makes it susceptible to overfitting.

Wang et al. tested and evaluated five neural network structures for OCT diagnosis [22] (DenseNet121, ResNet50, DPN92, ResNext101, CliqueNet), and VGG16, VGG19, inception-V3 neural networks, and support vector machine (SVM) methods were added in order to improve experimental comparisons. The network was fine-tuned using features extracted from the OCT dataset, and evaluation was carried out using two public datasets of 3231 and 5084 images, respectively. The dataset used for this experiment consists of eyeball images, not just retina images from OCT; thus, the pre-processing required for the screening of images and the size of the block is time-consuming, and training takes much longer.

Smitha et al. [23] introduced a GAN-based system for retinal disorder diagnosis in which the discriminator classifies the image into normal or abnormal categories. Their method employed denoising enhancement of the retinal layers as a pre-processing step. Two datasets were used for evaluation. Overall accuracy was 83.12% on a small dataset (3980 images: DME, dry AMD, and NORMAL) with low training parameters and 92.42% on a larger dataset (83,605 images: CNV, DME, NORMAL, and Drusen) with larger training parameters. The shortcomings of this method are that segmentation output greatly depends on the quality of the ground-truth images and that image denoising has a high probability of overfitting and thus does not enhance the generalization ability of the classifier. Tsuji et al. [24] constructed a network that utilized the capsule network to improve classification accuracy. Their architrave was built on six convolutional layers (CL) and one primary capsule network. Additionally, four CLs were added to the capsule network architecture of two CLs and one fully connected (FC) layer. Their method achieved an accuracy of 99.6%. The network requires a fixed-input image of 512×512. Resizing utilized linear interpolation, which causes some undesirable softening of details and can still produce somewhat jagged images.

In order to detect and grade the severity of DR, Reddy et al. [25] introduced a hybrid deep architecture that utilized a modified grey wolf optimizer with variable weights and attention modules to extract disease-specific features. The hybrid system aided in the joint DR–DME classification on the publicly available IDRiD dataset and achieved detection accuracy rates of 96.0%, 93.2%, and 92.23% for DR, DME, and joint DR-DME, respectively. Upadhyay et al. designed a cohesive CNN approach. The shallow-network (five-layered) layers were cohesively linked to allow for a smooth flow of image features, and batch normalization was instilled along with every activity layer. The approach obtained an accuracy of 97.19% for retinal disease detection for four-class classification [26]. A hybrid fully dense fusion CNN (FD-CNN) architecture was developed by Kayadibi et al. [27] to detect retinal diseases. They first employed a dual hybrid speckle reduction filter to diminish OCTs speckle noise followed by the FD-CNN to extract features. The classification was performed by deep SVM (D-SVM) and deep K-nearest neighbor (D-KNN) classifiers. The hybrid FD-CNN showed significant performance improvement compared to the single performance of CNN.

**Figure 1:**
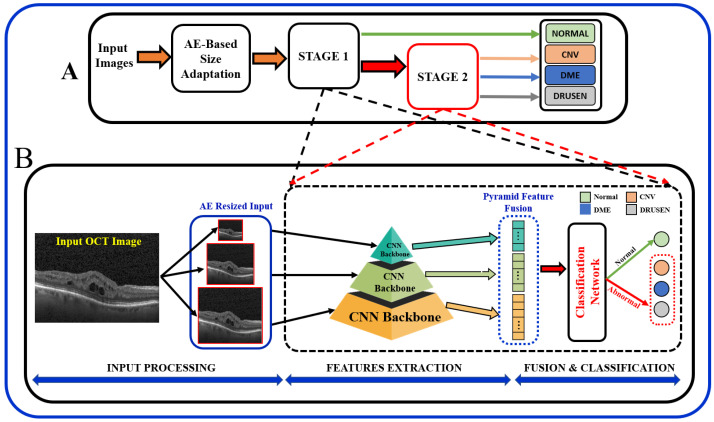
Schematic of the proposed multi-stage (**A**) and multi-resolution deep architecture model (**B**) for retinal disorders diagnosis using OCT scans.

In summary, the existing literature proposes various techniques, and it is important to note that the results of these papers vary depending on the specific task, dataset, and the DL technique used. Most of the existing literature used larger datasets while using pre-trained models, and some methods employed direct fusion for multi-scale predictions. Furthermore, features related to the higher-order reflectivity of the OCT images were not utilized in conjunction with deeper features, and cascaded classification was not investigated. This paper proposes a multi-stage classification of OCT image features that integrates discriminatory features through a multi-resolution feature pyramid with a scale adaptation module. The proposed cascaded multi-stage classification system is divided into two main steps (Figure 1). First, a scale adaptation network module is used to obtain various image scales for ensemble learning. Second, a transfer learning approach is utilized to extract features from OCT images using a pyramidal structure that allows for the extraction of differently scaled features from the same image dataset. Finally, the extracted features from three different scales of input images are fused to produce a single feature for classification. This fused feature has a rich concentration of local and global features at different levels. Using the one-vs.-rest (OVR) classifier, a binary classification of normal vs. abnormal (CNV, DME, or Drusen) is trained at the first stage, and the abnormal outputs are further passed through the same classification pipeline using different classifier algorithms to differentiate the classes in the second stage.

The main contributions of this work are as follows: (i) we designed a multi-scale, pyramidal, feature-rich input, as compared to single-scale, through the ensemble/fusion of multi-resolution features for classification; (ii) in order to extract prominent features from the input image, we adopted a scale-adaptive network architecture for generating the multi-scale input images instead of using image resizing; (iii) we utilized a transfer learning technique to extract the features in order to facilitate intermediate feature learning; (iv) we used a two-stage classification approach for a global (binary: normal vs. abnormal) and multi-disease classification overall pipeline fusing both lower- and higher-scale features; (v) we improved classification accuracy for both binary and multi-class scenarios using cross-validation despite the great overlap among the extracted features from the OCT images.

This manuscript is partitioned into four sections. An introduction to OCT and its role in retinal disease diagnosis in modern CAD systems is given in Section 1. This is followed by a relevant review of the recent literature work on this topic as well as the paper’s contributions. The materials and methods used along with specifics on the structure of the developed pyramidal architecture are fully detailed in Section 2. The dataset used, the employed performance criteria, the experimental design, the network parameters, the results, and a discussion are given in Section 3. At last, Section 4 provides work conclusions and limitations and future work suggestions.

## 2. Materials and Methods

In order to obtain better predictive performance, we developed a two-stage framework that includes pyramidal feature extraction, multiresolution feature ensemble, and classification. The input to the designed system is retinal OCT images obtained from two publicly available datasets. The proposed architecture provides both global (normal vs. abnormal) and stratified abnormal classifications. The proposed network architecture is schematized in Figure 1 with details described below.

OCT images that are collected from different imaging systems have different sizes, and using TL for the pre-trained network requires downscaling of the input images to fit the employed pre-trained model’s input. Unfortunately, downscaling exhibits the loss of important information from images. In order to account for this, we developed an autoencoder (AE)-based resizing module that accepts OCT images of any size and resizes them for use with pre-trained backbones when applying transfer learning. AE networks are considered unsupervised methods (no labels) that learn a latent-space (compressed) representation of the training data. The main advantage of AE neural architecture is its ability to filter out the noise and irrelevant information while reconstructing its output with minimal information losses. In our design, the AE module aims to resize the input images for use as an input in a pre-trained feature extraction ensemble architecture.

**Figure 2:**
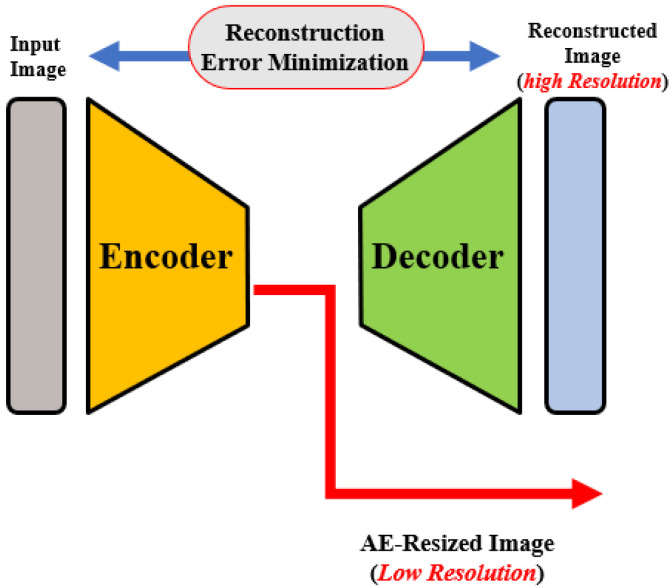
Illustration of the autoencoder-based size adaptation network.

The AE module is used to generate three different image scales for the proposed pyramidal feature extraction and ensemble learning (i.e., 224×224×3, 112×112×3 and 56×56×3). The module architecture is shown in Figure 2. The encoding path consists of consecutive convolution and pooling layers, which produce the feature map $F_{AE}$ of size 224×224×3. $F_{AE}$ is then processed through CL, transposed convolutional, and reshaped to 224×224×12. Original and processed $F_{AE}$s are integrated using the concatenation layer to produce both high and low-resolution images. The former is generated from $F_{AE}$ and is fed to the pyramidal feature extraction network. The latter is required for the module training phase in order to ensure that the reconstruction error between the module’s output and the original input image is minimal, i.e., the network learns important features from the inputs and discards redundancy and noise.

For AE module training, a custom loss that combines two pseudo-Huber loss functions and a log-cosh loss function for high resolution and low resolution, respectively, is used. Pseudo-Huber loss is more robust against outliers. Its behaviors for small and large errors resemble squared and absolute losses, respectively, and are defined mathematically as [28]:

(1) $${}_{P}Huber\left(x\right)=\delta^{2}\left(\sqrt{1+{\left(\frac{x}{\delta }\right)}^{2}}-1\right)$$

Here, *x* is the difference between the actual and predicted values and δ is a tunable hyper-parameter. On the other hand, the log-cosh loss function logcosh(x)=log(cosh(x)) is similar to Huber loss, but it is double differentiable everywhere [29]. Again, *x* is the difference between the actual and predicted values.

Following the AE-based resizing, the feature extraction step is performed for both global or binary (normal vs. abnormal) as well as for multiclass (CNV vs. DME vs. Drusen) classification of OCT images. At this stage, extraction of discriminating features from the retinal images is performed using pyramidal DL-based architecture. In order to achieve feature-rich classification as compared to single-level networks, a pyramidal DL system is proposed to extract various information to help with multi-class classification tasks; see Figure 1A. Namely, retinal images are resized using the AE module at three different scales (224×224, 112×112 and 56×56). Then, each of the pyramidal CNNs constructs a hierarchical representation of the input images that is then used to build a feature vector which in turn is eventually fused as a feature for the classification task. Although encoders in a wide variety of famous DL networks create a pyramidal feature that can be fused [18], the performance depends on fusion techniques. Thus, we chose to fuse the features of several networks in order to improve the semantic representation of the proposed model.

**Figure 3:**
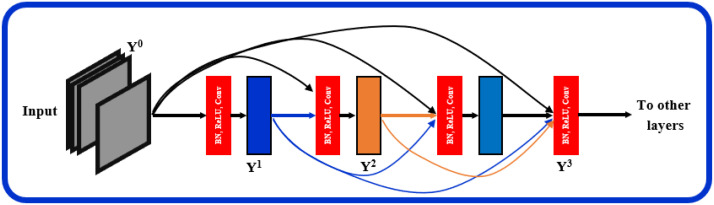
Layered dense block representing direct connections between layers.

The proposed architecture, Figure 1, can be seen as a multiresolution feature ensemble in which each CNN path utilizes transfer learning. Transfer learning is a great way to obtain significant results in a classification problem with low data volume. We adopted the pre-trained DenseNet201 model [30] in this work as the backbone of our pyramidal network. DenseNet has performed brilliantly on a variety of datasets and applications where direct connections from all previous layers to all following layers are established; Figure 3. This not only provides ease of training by facilitating feature reuse by different layers and improving connectivity but also increases the variance in later-layer inputs and thus enhances performance [31].

Dense blocks are formed in the network design for downsampling purposes and are separated by layers known as transition layers. The latter help the network to learn intermediate features and consists of batch normalization (BN), 1×1 convolution layers, and finally, a 2×2 average pooling layer. The BN stabilizes and speeds up the training process. A given feature map at layer *l* can be described mathematically as $Y^{\prime }=R_{1}\left(\left[Y^{0},Y^{1},\ldots \ldots ,Y^{1-1}\right]\right)$ where: $R_{1}$: is a non-linear transformation comprised of BN, a nonlinearity, and a convolution of 3×3. $\left[Y^{0},Y^{1},\ldots \ldots ,Y^{l-1}\right]$ refers to the feature map concatenation corresponding to layers 0 through (l−1) that are incorporated in a single layer.

Another hyperparameter, *k*, specifies the growth rate, or the rate at which the layer’s size in individual blocks of the network grows. It can be visualized as a regulator controlling the flow of information in successive layers to reach state-of-the-art results. For instance, when k=11, a filter size of 11 is used at each layer in an individual block. Generally, DenseNet performs well when smaller *k* are used, as the architecture considers feature maps as the network’s global state. As a result, each subsequent layer has access to all previous layers’ feature maps. Each layer adds *k* feature maps to the global state, with the total number of input feature maps at the *l*-th layer ${\left(\mathrm{FM}\right)}^{I}$ is defined as ${\left(\mathrm{FM}\right)}^{\prime }=k^{0}+k\left(l-1\right)$, where the channels in the input layer are determined by $k^{0}$.

**Figure 4:**
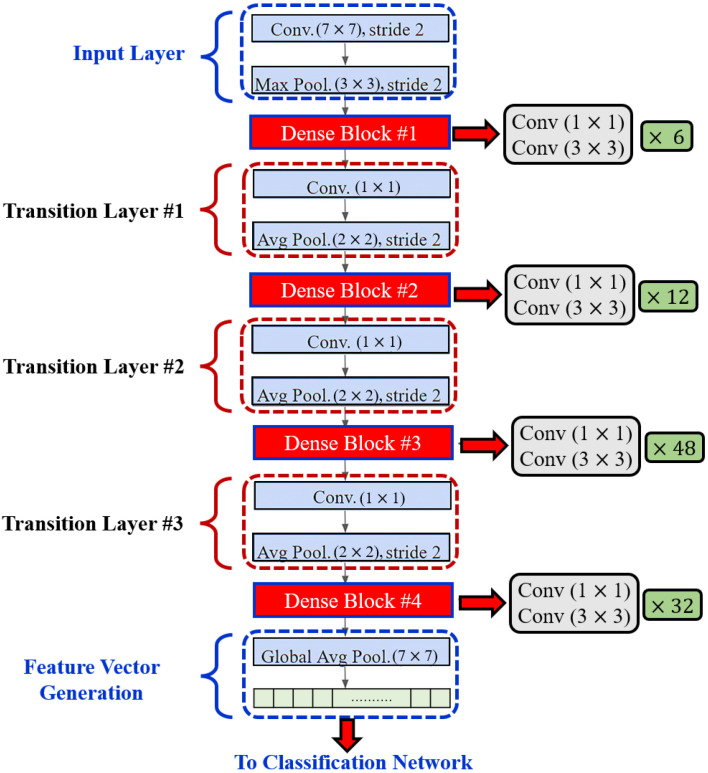
Layered Architecture of DenseNet201.

In order to enhance computational efficiency, a 1×1 convolution layer is added before each 3×3 convolution layer (see Figure 4) to reduce the number of input feature maps, which is often greater than the number of *k* output feature maps [32]. The global pooling layer pools the input features’ overall spatial locations at the end of each DenseNet path. The resulting vectors are then used to obtain the feature representations of the training and testing images and are fused for classification.

Finally, once all feature vectors for all three CNNs are constructed, they are fused (concatenated) to form predictor variables in a classification network. Features are extracted from pyramidal CNNs at the last layer just before the fully-connected layer. Since we used a pre-trained model, the number of features is typically fixed and is not affected by the input image size or other factors during inference. The size of the feature vectors for the three scales was 1920 individually (5760 after fusion). For classification, we used different classifiers in the first stage (binary) to classify the dataset into normal and abnormal as well as in the second stage (multiclass) to further differentiate the abnormal into three different classes. Namely, we used multilayer perceptron (MLP), logistic regression (LR), SVM, decision tree (DT), random forest (RF), and Naïve Bayes (NB) [33,34]. LR is a predictive analysis classifier that uses the Sigmoid function to predict input features and corresponding weight into a probabilistic output. SVM finds a hyperplane in N-dimensional space (N is a number of features) that distinctly classifies the data points of classes using the maximum margin. Although commonly used in data mining to reach a goal, DT is a supervised learning tree-structured classifier that predicts the value of a target variable by learning simple decision rules inferred from the data features. Similarly to DT, RF builds decision trees from various samples and takes the average to improve the predictive accuracy of that dataset. Finally, NB is a probabilistic ML classifier built on the Bayes theorem that predicts the probability of belonging to the “A” class given that “B” has occurred. The features are independent of each other, bringing about the name Naïve.

## 3. Experimental Results and Discussion

Evaluation to assess the proposed system is performed using various experiments on a UCSD dataset, and both binary and multi-class classification stages have been conducted. The first classification stage (binary) classifies the image as either a normal or abnormal retina, and the second (or the multi-class) stage stratifies the input image as either DME, CNV, or Drusen. The pyramidal CNNs were trained on publicly available datasets [19]. The dataset contains OCT images (Spectralis OCT, Heidelberg Engineering, Germany) from retrospective cohorts of adult patients provided by the Shiley Eye Institute of the University of California San Diego, the California Retinal Research Foundation, Medical Center Ophthalmology Associates, the Shanghai First People’s Hospital, and Beijing Tongren Eye Center [19]. About 108K OCTs in total for four classes (CNV: 37,206, DME: 11,349, Drusen: 8617, normal: 51,140) and the testing set containing 1000 retinal OCT images (250 from each class) are available from Reference [35]. We used Jupyter Notebook to implement the software on a Dell Precision 3650 Tower ×64-based workstation with an Intel Core(TM) eight-core CPU running at 2.50 GHz, 64 GB RAM, and with NVIDIA RTX A5000 GPU.

The multilayer perceptron (MLP) pyramidal networks were trained over 50 epochs with a batch size of 128. Additionally, a 5-fold cross-validation strategy was utilized as an unbiased estimator to assess the performance of our ensemble model against other methods. The use of cross-validation partially reduces problems of overfitting or selection bias and also provides insights on how deep architecture will generalize to an independent dataset. Both training and testing data were mixed and cross-validation was employed on the total dataset. All of the dense layers for both the first and second stages used the rectified linear unit (ReLU) as their activation function. Binary cross-entropy for the first stage and sparse categorical cross-entropy for the second stage were utilized as the loss function. An Adam optimizer was employed with a learning rate starting at 0.001, and this was reduced automatically during the training phase in order to improve results whenever the loss metric had stopped improving on both stages. Total network parameters of 1,665,197 out of 1,665,397 parameters were used for training in the first stage and 3,041,711 out of 3,041,911 for the second stage.

We first investigated the first stage for the global (i.e., binary) classification of the retinal images as normal or abnormal. This step mimics human perception of separate groups. Evaluation of the proposed pipeline performance is conducted using known classification metrics, such as accuracy, sensitivity, specificity, and AUC of the receiver operating curve (ROC). Those metrics are defined in terms of experiments’ outcomes of true positive (TP), true negative (TN), false positive (FP), and false negative (FN) as follows:

(2) $$\mathrm{Acc}=\frac{\mathrm{TN}+\mathrm{TP}}{\mathrm{TN}+\mathrm{TP}+\mathrm{FN}+\mathrm{FP}},\mathrm{Sen}=\frac{\mathrm{TP}}{\mathrm{TP}+\mathrm{FN}},\mathrm{and}\;\mathrm{Spc}=\frac{\mathrm{TN}}{\mathrm{TN}+\mathrm{FP}}$$

Different ML classifiers were further employed for both stages, and our overall MLP model accuracy performance for both stages is demonstrated in Table 1 for the 5 folds. For the ML classifiers, default parameters were used for the classification. SVM (kernel = ‘rbf’ and decision function = ‘OVR’), DT (criterion = ‘gini’, splitter = ‘best’, none for others), RF (criterion = ‘gini’, estimator = ‘100’), NB (priors = ‘none’, smoothing = ‘1e-9’) but for LR (solver = ‘liblinear’).

**Figure 5:**
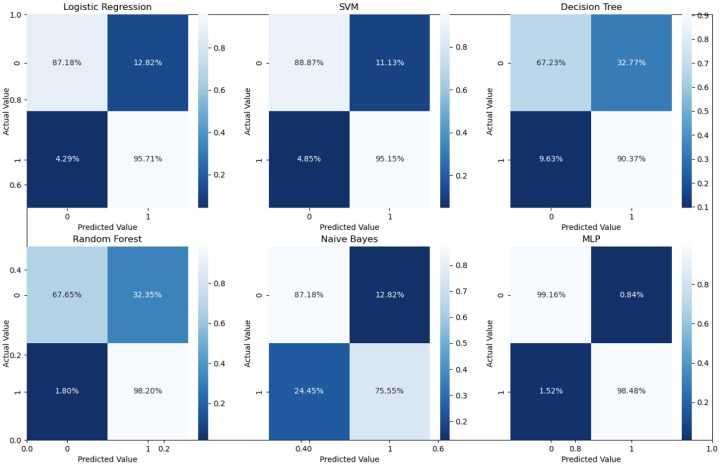
Confusion matrices for the first stage using 5-fold cross validation on the UCSD dataset.

As can readily be seen, MLP performed best (97.79% accuracy in the first stage and 96.83% in the second stage) among the other classifiers. This is mainly due to its capability to learn complex nonlinear patterns by amplifying relevant aspects of input data and suppressing irrelevant information [36]. Additionally, confusion matrices were used as an alternative quantitative evaluation. Figure 5 shows our network’s confusion matrix for different classifiers in the first stage using 5-fold cross-validation. Network evaluation and monitoring benefit from confusion matrices. From the obtained confusion matrix, other indices such as precision, f1 score, and recall can be derived. For the assessment evaluation of classification models, both the confusion matrix and related metrics are typically employed together.

**Figure 6:**
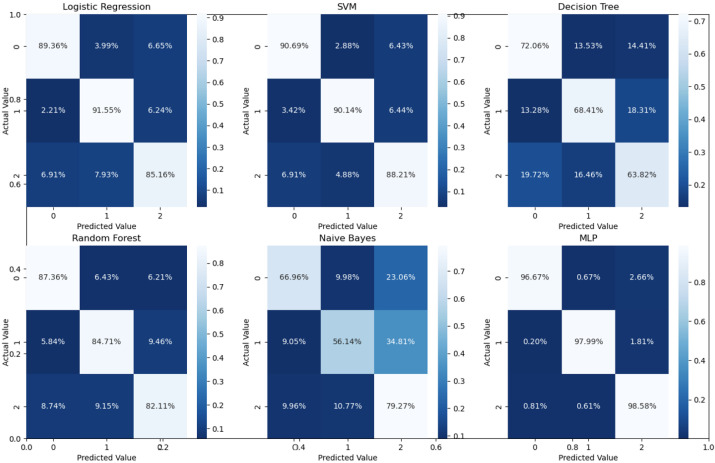
Confusion matrices for different classifiers for the second stage (i.e., three classes using 5-fold cross-validation on UCSD data set.

**Figure 7:**
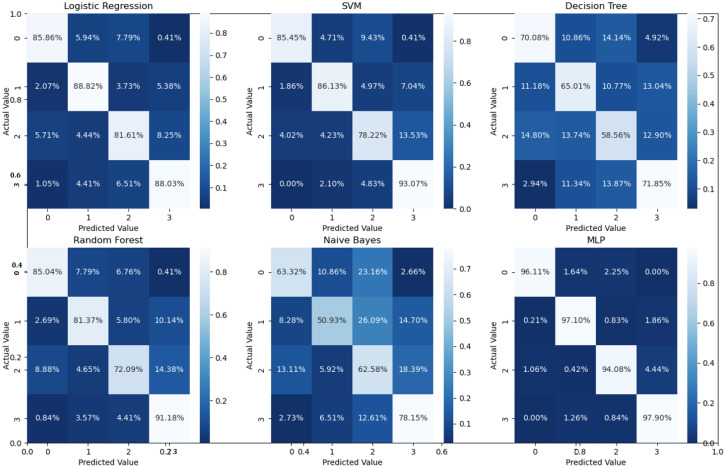
Confusion matrices for the four classes using 5-fold cross-validation on the UCSD dataset.

**Figure 8:**
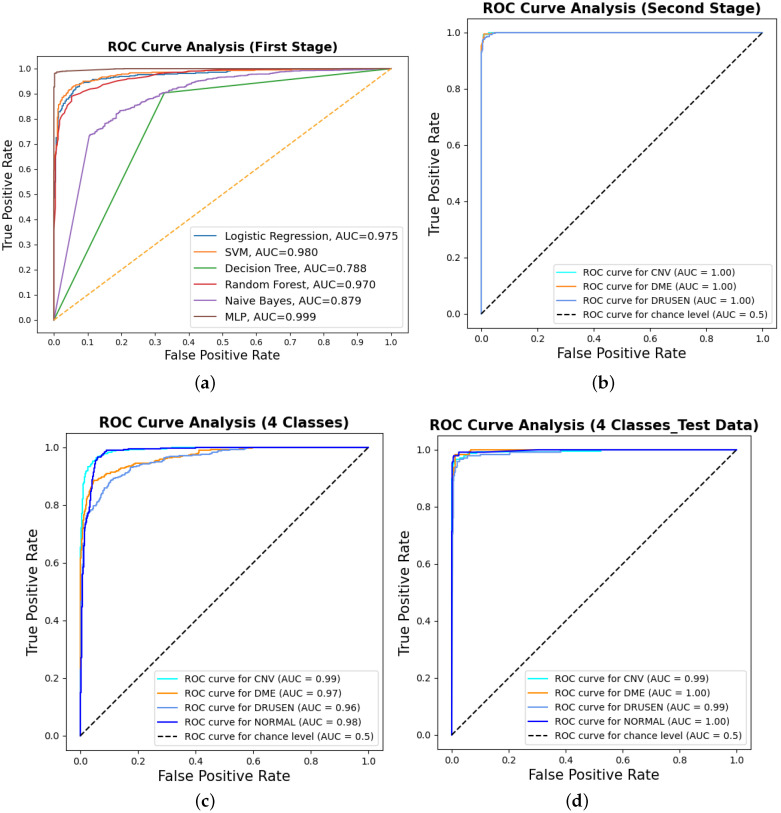
The receiver operating characteristic (ROC) curves for the proposed cascaded framework using cross-validation on the UCSD dataset: (**a**) binary classification using different classifiers; (**b**) second-stage classification OVR using the MLP. Furthermore, the figure shows the ROCs for all-at-once four-class classification using the MLP for (**c**) cross-validation and (**d**) test dataset only.

Binary classification is an initial step in any treatment procedure by retina specialists. However, personalized medicine would require the determination of the disease and, more appropriately, its grade. Thus, the second set of experiments investigated multi-class classification (DME vs. CNV vs. Drusen). The results for different classifiers are summarized in the middle part of Table 1, and the second stage confusion matrices are depicted in Figure 6. Moreover, in order to demonstrate the efficacy of the pipeline to separate the four classes, we performed an additional experiment using cross-validation on the UCSD dataset. The model accuracy using the evaluation metrics is given in the right part of Table 1, and the confusion matrices are given in Figure 7. Besides accuracy metrics, the system’s accuracy and robustness are confirmed using the receiver operating characteristics (ROC) curves in Figure 8. The figure depicts the ROCs for the proposed cascaded classification network for the first stage (Figure 8a), the second stage (Figure 8b), and all-at-once classification (Figure 8c).

According to Table 1 and the confusion matrices in Figure 5, Figure 6 and Figure 7, binary classification demonstrated the highest accuracy compared with the second stage and all-at-once classification. This is an important aspect of the presented cascaded classification structure that aligns with clinical diagnostics and emulates the process of a physician’s diagnosis. Specifically, the system is designed to initially classify patients into broad groups with a high level of confidence, such as distinguishing between normal and abnormal cases or identifying AMD versus DME. Once patients have been stratified and critical cases have been identified, physicians can then conduct a more comprehensive evaluation using other available clinical signs and biomarkers. This allows for a refined differential diagnosis, moving beyond OCT-based signs alone and towards an accurate and specific diagnosis. Although there is the recent advantage of multi-scale DL-based fusion workflows in many applications, including retinal applications, separating a large number of classes (sub types or grades) at once is a challenging task. This explains the slight reduction in accuracy when the system separates all four classes at once. This, however, can be enhanced in practice by integrating other available clinical signs/biomarkers/images for challenging and complicated retinal diseases, including other diseases.

Our ultimate goal was to design and evaluate a versatile system that can be extended to detect various retinal diseases. In order to explore the benefits of TL, we conducted an additional experiment in which we evaluated several well-known ImageNet-based pre-trained feature extractor architectures as replacements for DenseNet201. The architectures we tested included VGG16, VGG19, Xception, and InceptionV3. The features extracted from these architectures were then fused and used for classification. The results of this experiment are presented in Table 2. The accuracy of the different backbones showed slight variations, with the VGG architectures performing particularly well. These findings demonstrate the potential of our cascaded architecture to leverage various pre-trained models, which can be further improved through fine-tuning. Consequently, our system can be extended to detect other retinal diseases not covered by the datasets used in this study.

**Figure 9:**
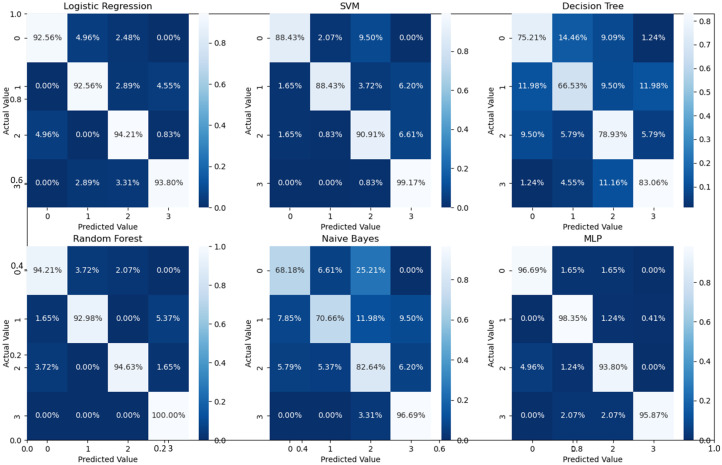
Confusion matrices for different classifiers for the four classes using UCSD test data only.

All of the above experiments employed cross-validation for the cascaded as well as all-at-once classifications for the four categories in the UCSD dataset. In addition to that, we have further conducted an additional experiment for four-class classification using the train/test data split of the UCSD dataset. The overall accuracies, confusion matrices, and ROCs for the examined classifiers for the four-class classification on the test dataset are given in Table 3, Figure 8d and Figure 9. The results are consistent with the results in Table 1 with a slight accuracy increase of 2%.

Moreover, the advantage of our system for retinal diseases’/disorders’ diagnosis has been compared with standard and recent literature methods. All of the compared networks were tested on the available images in order to compare their abilities for both the multi-class and binary stages. For the first-stage classification, our network performance was compared with traditional methods pre-well-trained on the Imagenet dataset [37] mainly to show the effect of the ensemble learning and scale adaptation network on the overall performance. The comparison included the DenseNet121 by Huang et al. [30], the ResNet101 by Szegedy et al. [38], and the method by Haggag et al. [39], which was designed for retinal image analysis. Since the UCSD dataset does not have ground truth for the retinal layers to compute other local and global feature images, we only used the grayscale images in Reference [39]. For the pre-trained network, the top layer was removed and replaced by a fully connected layer with a dropout of 40% and a final node of the sigmoid activation function for classification. A summary of the performance metrics is given in Table 4. Statistical significance tests were performed using a paired Student’s *t*-test to assess the accuracy of the proposed method in comparison to the other methods. The results indicated that our method is statistically significantly better than the compared methods (*p*-value < $10^{-4}$). Further, an ablation experiment was conducted to verify the effect of the scale adaptation module on the classification performance. For the first- and second-stage classification, our network showed and average accuracy of 95.76% and 94.93%. The overall enhancement (∼2%) was promising, and future work should be conducted to explore other module improvements.

For the four-class comparison, our architecture was compared with well-known CNN models and multiple well-known classification frameworks that reported accuracy on the UCSD dataset. The comparative accuracy is demonstrated in Table 5, and the confusion matrices for the different classifiers are shown in Figure 7. As can readily be seen in Table 1 and Table 5, the proposed pipeline showed improved performance compared to its counter and off-the-shelf networks. This is also confirmed using Student’s *t*-test, (*p*-values < $10^{-4}$) similar to the binary classification.

To verify our system performance on other datasets in addition to the UCSD dataset, we tested our approach on the Duke dataset [40], which contains a total of 3231 OCT images for three classes: normal (1407), AMD (723), and DME (1101) patients. The dataset does not have any training and testing splits, so we followed the same approach as was used by Kayadibi et al. in [27], where the train–test split was 90% and 10%, respectively. The proposed pyramidal cascaded architecture results compared with other methods tested on the same dataset are given in Table 6. The results document the better performance of our architecture. These results are encouraging, and we ultimately plan to expand our system in future work to be able to be even more specific, such that we identify not purely signs (e.g., macular edema or CNV), but could actually distinguish between different causes of cystoid macular edema (CME) based on OCT features, such as retinal vein occlusion, diabetic macular, or uveitic macular edema.

**Table 4:** Comparisons with other related work for binary classification on the UCSD data set.

Method	Acc%	Sen%	Spc%
Haggag et al. [39]	90.1	87.7	92.61
Huang et al. [30]	92.30	89.01	94.61
Szegedy et al. [38]	89.12	82.3	85.18
Proposed	97.79	95.55	99.72

**Table 5:** Comparisons with other related work for four-class classification using 5-fold cross-validation.

Applied Method	Acc%	Sen%	Spc%
Fang et al. (JVCIR) [41]	87.3	84.7	95.8
Fang et al. [42]	90.1	86.8	96.6
S.-Paima et al. [18]	93.9	93.4	98.0
Proposed	94.3	96.3	98.7

**Table 6:** Overall accuracy in comparison with other works tested on the Duke data set.

Applied Method	Acc%	Sen%	Spc%
Thomas et al. [43]	96.66	—	—
Amaladevi and Jacob [44]	96.20	96.20	99.89
Kayadibi and Güraksın [27]	97.50	97.64	98.91
Proposed	99.69	99.71	99.87

## 4. Conclusions

We have developed a multi-level, multi-resolution feature ensemble architecture for the classification of retinal disorders. The proposed pipeline mimics the human perception of global diagnosis followed by stratification of the suspected cases. The scale-adaptation networks help to produce multi-scale inputs while retaining valuable information when downscaling. Additionally, the pyramidal layout helps extract various information to help with the binary and multi-class classification stages of the three retinal disorders. In summation, the proposed architecture not only provides global diagnosis but also automatically distinguishes between different retinal diseases, thus allowing for earlier treatment of the patient’s condition. Despite promising results, some limitations of this work should be addressed in future work. First, the proposed system should be evaluated on more challenging retinal datasets with different diseases for rigorous evaluation. Second, we used only pre-trained CNNs for feature extraction, and thus, more evaluation using visual transformers should be investigated.

Future research venues will explore integrating the architecture into more-complex retinal disorders’ pipelines to include, for example, sub-grades of disease (such as dry and wet AMD) for accurate and precision medicine. Further, integration of explainable AI modules (e.g., Grad-CAM, LIME, etc.) to gain further insights into the reasoning behind the systems’ output will be explored. Finally, a weighted fusion of the multi-scale features will be thoroughly investigated as well as the study of additional higher-order features using spatial models.

## Figures and Tables

**Table 1:** Performance of different classifiers for the proposed cascaded classifications all well as for all-at-once (four classes) classification using 5-fold cross validation on the UCSD dataset. LR: logistic regression; SVM: support vector machine; DT: decision tree, RF: random forest; NB: Naïve Bayes, and MLP: multilayer perceptron.

	First Stage (Binary)				Second Stage (3-Classes)			4-Classes		
Classifiers	Acc%	Sen%	Spc%	AUC%	Acc%	Sen%	Spc%	Acc%	Sen%	Spc%
MLP	97.79	95.55	99.72	99.86	96.83	97.75	98.87	94.26	96.29	98.74
LR	89.23	87.00	95.77	97.47	89.34	88.69	93.99	85.95	86.08	94.91
SVM	90.33	85.80	96.29	97.98	89.47	89.68	94.56	86.53	85.72	94.79
DT	80.14	69.72	89.32	78.80	69.92	67.15	81.67	65.22	65.55	85.15
RF	85.40	92.53	90.20	97.04	84.62	84.57	91.61	81.10	80.11	92.82
NB	73.71	54.04	94.70	87.90	67.46	67.46	81.00	63.82	63.75	84.35

**Table 2:** Performance of different feature extractors for the proposed cascaded classifications all well as for all-at-once (four classes) classification using 5-fold cross-validation on the UCSD dataset and multilayer perceptron.

	First Stage (Binary)			Second Stage (3-Classes)			4-Classes		
Classifiers	Acc%	Sen%	Spc%	Acc%	Sen%	Spc%	Acc%	Sen%	Spc%
Xception	95.91	98.96	95.64	91.96	96.86	98.43	93.15	97.97	99.32
InceptionV3	95.34	89.16	99.50	92.21	95.64	97.80	91.76	94.24	98.01
VGG19	95.94	97.57	99.31	93.88	95.40	97.67	93.39	97.01	99.67
VGG16	97.26	98.55	99.99	93.92	96.15	99.58	94.65	99.16	96.72
DenseNet201	97.79	95.55	99.72	96.83	97.75	98.87	94.26	96.29	98.74

**Table 3:** Four-class classification performance using the UCSD test dataset only. LR: logistic regression; SVM: support vector machine; DT: decision tree, RF: random forest; NB: Naïve Bayes; MLP: multilayer perceptron.

	Metric		
Classifier	Acc%	Sen%	Spc%
MLP	96.17	96.17	98.69
RF	95.45	94.83	98.22
LR	93.28	93.29	97.66
SVM	91.73	95.97	98.63
DT	75.92	78.51	91.64
NB	79.54	79.55	92.23

## Data Availability

The datasets are publicly available at https://data.mendeley.com/datasets/rscbjbr9sj (accessed on 1 February 2023) and https://people.duke.edu/~sf59/Srinivasan_BOE_2014_dataset.htm (accessed on 1 Febuary 2023).
